# An elevated neutrophil-to-lymphocyte ratio associates with weight loss and cachexia in cancer

**DOI:** 10.1038/s41598-020-64282-z

**Published:** 2020-05-05

**Authors:** Tyler Barker, Gail Fulde, Bryce Moulton, Lincoln D. Nadauld, Terence Rhodes

**Affiliations:** 1 Precision Genomics, Intermountain Healthcare, St. George, UT 84790 USA; 2 Department of Nutrition and Integrative Physiology, University of Utah, Salt Lake City, UT 84112 USA; 3 School of Medicine, Stanford University, Stanford, CA 94305 USA

**Keywords:** Cancer, Immunology

## Abstract

Systemic inflammation is present during and serves as a diagnostic tool for cancer-associated cachexia and is detrimental to serum 25-hydroxyvitamin D (25(OH)D) concentrations in non-cancer conditions. The neutrophil-to-lymphocyte ratio (NLR) is a desirable measure of systemic inflammation because it is easily calculated from a routine complete blood cell count with differentials. We sought to determine if an elevation in the NLR associates with greater weight loss, cachexia, and lower serum 25-hydroxyvitamin D (25(OH)D) concentrations in patients with advanced cancer. Advanced colon, lung, and prostate cancer patients (stages III/IV; n = 50) were retrospectively studied and separated into one of two groups: 1) Above (n = 25) or 2) Below (n = 25) the median NLR of 3.15 determined at diagnosis. Around the time of diagnosis, serum 25(OH)D and body weight were assessed, while body weight was assessed again at a later date. Weight loss and cachexia were significantly (both p < 0.05) greater and there was a trend (p < 0.10) for lower serum 25(OH)D concentrations in the Above group. We conclude that an elevation in the NLR associates with greater weight loss and cachexia, and potentially, a lower serum 25(OH)D concentration in patients with advanced colon, lung, or prostate cancer.

## Introduction

Systemic inflammation is a key determinant of host defenses and repair processes, but an excessive response is detrimental to essential physiological processes and mortality. Fatigue, appetite loss, reduced dietary intake, poor quality of life, and inferior overall survival are common symptoms of cancer and disease progression that associate with diverse biomarkers of systemic inflammation^1–7^. The neutrophil-to-lymphocyte ratio (NLR) is an attractive biomarker of systemic inflammation because it is easily calculated from the absolute neutrophil and lymphocyte counts obtained from a routine complete blood cell count with differentials. This readily available biomarker of systemic inflammation predicts survival independent of tumor or disease stage in various cancers^8^ and associates with cancer-related cachexia^9^.

Cachexia is a multi-factorial syndrome associated with an underlying disease and characterized by the loss of skeletal muscle mass with or without the concomitant loss of fat mass^10^. Cachexia is a devastating syndrome because, in part, it is linked to a reduced response to anti-cancer therapy, diminished quality of life, and inferior overall survival^11,12^. Approximately 20% of all cancer-related deaths are attributed to cachexia^13^ and reversing cachexia prolonged survival in experimental animals^14^. Although data identifies a concurrent increase during and the diagnostic role of systemic inflammation in cachexia^7,10,15^, very few studies have reported if systemic inflammation associates with weight loss and cachexia in patients with advanced cancers.

Low or deficient circulating 25-hydroxyvitamin D (25(OH)D) concentrations are highly prevalent in advanced cancer patients with cachexia^16^ and mediate skeletal muscle wasting or loss in experimental animals^17–19^. Serum 25(OH)D is the major metabolite used to determine vitamin D status, and it is plausible that fatigue, poor appetite, anorexia, low sunlight exposure, and the reduced ability to absorb dietary vitamin D may contribute to low circulating 25(OH)D concentrations in patients with cachexia. Provocatively, evolving data identifies inflammation as a proposed moderator of vitamin D metabolism. Specifically, in isolated peripheral blood mononuclear cells, diverse cytokines and cytokine exposure protocols mediate a 25(OH)D decrease^20,21^. Data from our lab and others illustrate a decrease in serum 25(OH)D concentrations during acute-systemic inflammation immediately following major (i.e., total knee arthroplasty) and minor (i.e., anterior cruciate ligament reconstruction) orthopedic surgeries^22,23^. Although low serum 25(OH)D increased systemic inflammation in patients with prostate cancer^24^, it is unknown if systemic inflammation associates with low serum 25(OH)D concentrations in patients with advanced cancer. Identifying the detrimental influence of systemic inflammation on serum 25(OH)D concentrations could reveal a new therapeutic target intended to protect against low vitamin D and possibly poor outcomes (i.e., disease progression and mortality) associated with low vitamin D in cancer.

Cachexia and low vitamin D are commonly associated with systemic inflammation, but conversely, it is unknown if an elevation in systemic inflammation associates with greater weight loss, cachexia, and low vitamin D. Based on this aforementioned gap in our knowledge, the purpose of this investigation was to identify if an elevated NLR associates with weight loss, cachexia, and serum 25(OH)D concentrations in cancer. We hypothesized that an elevation in the NLR associates with greater weight loss, cachexia, and lower serum 25(OH)D concentrations in patients with advanced cancer.

## Materials and Methods

Newly diagnosed (between January 1^st^, 2015 and December 31^st^, 2016) adult patients with advanced colon, lung, or prostate cancer (stages III or IV) were retrospectively studied at a single institution (Intermountain Healthcare, Salt Lake City, UT USA). Cancers were confirmed by standard histopathology methods. We limited this study to colon, lung, and prostate cancer patients in an attempt to include a proportional distribution between cachectic and non-cachectic patients. Based on previous reports, we anticipated approximately 50% of the targeted cancer patients to develop cachexia^25^.

Patients were initially identified using the International Classification of Diseases 9^th^ and 10^th^ Revision codes. Initial queries identified 100 subjects with serum 25(OH)D concentration data. However, 50 subjects were excluded due to random-missing data; such as lacking blood chemistries (i.e., neutrophil and/or lymphocyte counts), overall survival data, or treatment was performed at a different institution. The final analysis consisted of 50 subjects with colon, lung, or prostate cancer (Table 1). This study was approved with a consent waiver by the Institutional Review Board at Intermountain Healthcare.

**Table 1:** Results Below and Above the median NLR of 3.15.

	Median NLR	
	Below	Above
n (m/f)	25 (16/9)	25 (16/9)
**Disease site, n**		
*colon*	6	10
*lung*	11	9
*prostate*	8	6
Neutrophils, K/μL	3.80 (1.70)	6.50 (3.28)^a^
Lymphocytes, K/μL	1.90 (0.93)	1.20 (0.85)^a^
NLR	2.06 (0.74)	4.76 (2.60)^a^
age, y	72.0 (6.25)	74.0 (15.8)
height, cm	170 (17)	175 (19)
**Body weight, kg**		
*first*	83.9 (27.9)	87.1 (26.1)
*second*	81.7 (23.1)	81.7 (22.3)
Cachexic, n (%)*		
*no*	22 (88%)	14 (56%)
*yes*	3 (12%)	11 (44%)
days between body weight measures	172 (215)	202 (256)
**BMI, kg/m**^**2**^		
*first*	28.7 (6.7)	27.1 (8.5)
*second*	29.5 (6.4)	27.9 (7.1)
Deceased, n (%)^b^	5 (20.0%)	8 (33.3%)
Overall survival, d^b^	552 (435)	571 (296)
**Vitamin D status, n (%)**		
*Deficient*	2 (8%)	4 (16%)
*Insufficient*	2 (8%)	4 (16%)
*Sufficient*	21 (84%)	17 (68%)

Data presented as median (interquartile range) unless otherwise noted.

^a^p < 0.05 vs Below.

*p < 0.05.

^b^Excluding the subject in the Above group that expired due to head trauma.

Data was extracted from electronic medical records and time-aligned to diagnosis and treatment. Absolute neutrophil and lymphocyte counts were obtained around the time of diagnosis and prior to any cancer-specific treatment. After extracting the absolute counts and calculating the NLR, subjects were separated into one of two groups: (1) Above (n = 25) or (2) Below (n = 25) the median NLR of 3.15.

Total serum 25(OH)D concentrations (sum of D_2_ and D_3_; ng/mL) were determined using a chemiluminescent immunoassay and results were extracted from the electronic medical records database. Subjects were classified as vitamin D deficient, insufficient, or sufficient based on a serum 25(OH)D concentration ≤20, 21–29, or ≥30 ng/mL, respectively^26^.

Body weights were extracted from two time points: 1) the first available record around the date of diagnosis and 2) the next available record with a minimum of 14-d separating assessments. Cachexia was defined as a body weight loss >5% or >2% in patients with an initial body mass index <20 kg/m^2^ ^10^. Overall survival was determined as the length of time (d) from diagnosis that the patient was alive with a minimum follow-up of 1-year.

In order to detect a 5.0% difference in weight loss among means with a statistical power of 0.80 and at an α 0.05, at least 20 subjects were required per group, while in order to detect 3.02 ng/mL difference in serum 25(OH)D among means with a statistical power of at least 0.80 and at an α = 0.05, a minimum of 22 subjects per group were required. Data were checked for normality with a Shapiro-Wilk test prior to statistical analysis. For normally distributed data, statistical significance was assessed with a one-way analysis of variance. A Mann-Whitney U test was performed to assess the statistical significance between variables that were non-normally distributed. Categorical variable differences between groups were evaluated using separate Pearson chi-squared tests. Overall survival was assessed with a Kaplan-Meier analysis. Associations between variables were assessed with a Pearson Product Moment Linear or Spearman Rank correlation analysis. Significance was set a p < 0.05. All statistical analyses were performed with SYSTAT (version 13.1, Chicago, IL USA). Data presented as mean (standard deviation) or median (interquartile range) unless otherwise noted.

## Results

### Subject characteristics

Neutrophil counts were higher (p < 0.05), lymphocyte counts were lower (p < 0.05), and the NLR was elevated (p < 0.05) in the Above group (Table 1). Despite differences in hematological parameters, general subject characteristics (i.e., gender, cancer site, age, and height) were not significantly different above compared to below the median NLR.

### Weight loss and cachexia

**Figure 1:**
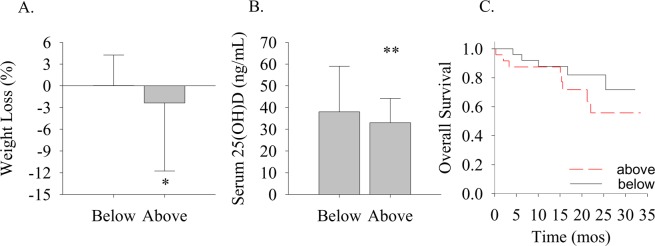
Weight loss, serum 25(OH)D concentrations, and overall survival between NLR groups. (**A**) Body weight loss (%) was significantly (*p < 0.05 vs Below) decreased in the Above compared to the Below group. (**B**) Serum 25(OH)D concentrations (ng/mL) tended (**p < 0.10 vs Below) to decrease in the Above compared to the Below group. (**C**) Overall survival (mos) was not significantly different between NLR groups (Below vs Above). Data presented as median (interquartile range).

Body weights and body mass index (BMI) were not significantly different between NLR groups (Table 1). However, weight loss (%) was significantly greater (p < 0.05) in the Above compared to the Below group (Fig. 1A). Furthermore, 44% in the Above compared to only 12% in the Below group developed cachexia (p < 0.05, Table 1). Although variability was apparent in the number of days separating body weight assessments (*see* Table 1), the number of days between body weight assessments was not significantly different between groups and the percent loss in body weight was not significantly correlated (r_s_ = 0.01, p = 0.97, data not shown) with the number of days between body weight assessments. Thus, in this short-term follow-up study, weight loss did not appear to be dependent on the time from diagnosis to the next body weight assessment.

### Serum 25(OH)D and vitamin D status

Most of the serum 25(OH)D concentrations were assessed after diagnosis but before treatment (Supplemental Table). Due to the time-based variability in available concentrations, we evaluated if serum 25(OH)D concentrations were significantly different before compared to after diagnosis and treatment. Despite temporal discrepancies in assessment, serum 25(OH)D concentrations were not significantly different before compared to after diagnosis or treatment (Supplemental Table). Consequentially, serum 25(OH)D concentrations were pooled and analyzed independent from date of collection.

Serum 25(OH)D concentrations tended (p < 0.10) to be lower in the Above compared to the Below group (Fig. 1B), while vitamin D status classifications were not significantly different between NLR groups (Table 1). An unexpected observation, however, was the number of subjects with a sufficient serum 25(OH)D concentration. Specifically, 84% in the Below and 68% in the Above groups possessed a sufficient vitamin D status. As a function of vitamin D status, the NLR was not significantly different between vitamin D status groups (Supplemental Fig.).

### Overall Survival

Medical records revealed head trauma as the cause of death for one subject in the Above group. To assess the potential confounding influence of non-cancer-related mortality on the study outcomes, we subsequently excluded the head trauma subject from a secondary analysis. Results between the NLR groups were not significantly different with compared to without the inclusion of the subject that expired due to head trauma. Therefore, the subject that expired due to head trauma was included in the final analysis and reported herein but excluded from the analyses comparing expiration and overall survival between NLR groups. The number of deceased subjects (Table 1) and overall survival (Table 1 and Fig. 1C) were not significantly different between NLR groups.

## Discussion

In this investigation, we provide novel evidence that an elevation in the NLR associates with subsequently greater weight loss and cachexia without altering overall survival in patients with advanced colon, lung, or prostate cancer. Additionally, there was a trend for lower serum 25(OH)D concentrations to associate with an elevation in the NLR. These data suggest that greater weight loss, cachexia, and potentially, lower serum 25(OH)D concentrations accompany or follow an elevation in NLR.

Systemic inflammation is a response to cancer-associated cachexia^9,15^ and deservedly implemented in the definition and diagnosis of cachexia^10,27^. Cancer-related cachexia or weight loss associates with various biomarkers of systemic inflammation, including but not limited to, select cytokines (e.g., interleukin (IL)–6 and IL-8) and C-reactive protein (CRP) concentrations^9,15,28,29^. Unfortunately, cytokine and CRP assessments are not routine in the clinic or clinical practice for cancer patients, and thus, challenge their practicality to assist in the daily physician decision process. Therefore, this study extends previous results^9,28,29^ by providing unique data that a biomarker of systemic inflammation easily calculated from a routine, standard of care clinical chemistry associates with weight loss and cachexia in cancer.

In multiple cancer types, the NLR is significantly elevated with cachexia compared to non-cachectic patients with advanced cancer (stages III and IV)^9^, suggesting a cachexia-driven inflammatory response. Although cachexia may mediate an increase in systemic inflammation, alternatively, inflammation is more likely contributing to cachexia^30^. Consistent with this latter theory, body weight changes have been found to inversely associate with the NLR in patients with non-small cell lung cancer^31^, implying an increase in weight loss with increasing systemic inflammation. Thus, the findings in the investigation corroborate and extend previous results in non-small cell lung cancer by demonstrating that an elevation in the NLR associates with greater weight loss and cachexia in patients with advanced colon, lung, or prostate cancer. Chronic or sustained inflammation may also compromise immune function and nutrition status^32^, and as suggested elsewhere, lead to poor clinical outcomes^33^.

The NLR alone or mathematically-integrated into a cachexia-index formula associates with or predicts poor outcomes in cancer patients, including inferior overall survival^34,35^. However, the NLR and overall survival were not associated in this study and is possibly related to a relatively small sample size^36^. In a meta-analysis, Templeton and colleagues^36^ provide strong evidence indicating a negative publication bias in small sample size studies examining the association between the NLR and overall survival. Additionally, the present study was limited to a 1-year follow-up, and therefore, it is reasonable to presume that a longer follow-up could illuminate a significant relationship between the NLR and mortality that is consistent with prior reports. It is also plausible that superior prognostic indices exist in the circulation other than the NLR, such as the platelet-lymphocyte ratio^37^, that associate with survival in cancer patients that were not examined in this study.

Serum 25(OH)D concentrations tend to decrease with an elevation in the NLR, which is somewhat consistent with the premise that inflammation moderates a serum 25(OH)D decrease^22,23^. In previous studies, diverse cytokines have been found to regulate the enzymatic machinery of vitamin D metabolism in isolated peripheral blood immune cells^20,21^, which could contribute to the decrease in serum 25(OH)D during inflammation *in vivo*^23^. Conversely, the more widely recognized relationship is an increase in inflammation as a result of a low serum 25(OH)D concentration^38^. Although differentiating whether inflammation induces a decrease in serum 25(OH)D or if low serum 25(OH)D mediates an increase in inflammation is beyond the scope of this study, it is noteworthy that low serum 25(OH)D does not associate with an increase in the NLR (*see* Supplemental Fig.) while an elevation in the NLR tends to associate with lower serum 25(OH)D concentrations (*see* Fig. 1B). Although these findings neither support nor refute the study hypothesis, the data does identify the necessity for additional research investigating the bidirectional relationship between inflammation (i.e., cytokines) and vitamin D in cancer.

Previous results indicate that approximately 50% of cancer patients display vitamin D deficiency (i.e., serum 25(OH)D ≤ 20 ng/mL) and 70% with vitamin D insufficiency (i.e., serum 25(OH)D < 30 ng/mL)^16^. In contrast, only 12% of the subjects had a serum 25(OH)D concentration ≤ 20 ng/mL and 24% of the subjects possessed a serum 25(OH)D < 30 ng/mL in the present study. In addition to inflammation, serum 25(OH)D concentrations are influenced by a variety of factors, including dietary, environmental, demographic, and genetics^38^; all of which, possess a regulatory influence on systemic inflammation and cancer. Therefore, the resulting serum 25(OH)D concentrations in this report could be regulated by a multitude of factors and potentially confound the association between inflammation and vitamin D. Nevertheless, the reason for such dramatic differences in vitamin D status between those previously and those herein is unclear and requires further research for later resolve. Considering the potential influence of vitamin D status or supplementation on cancer-related mortality, it is also plausible that the predominance of vitamin D sufficiency confounded the relationship between the NLR and overall survival.

In addition to those mentioned above, there are some limitations to this study that require further discussion. First, and although not statistically significant, body weight initially appeared to be slightly increased in the Above compared to the Below group. To account for this unexpected observation, body weight changes were normalized to the first assessment and analyzed as the percent change as opposed to the absolute (i.e., kg) loss in body weight. Second, body weight was initially assessed at the first available record around the date of diagnosis. Therefore, it is unknown if significant weight loss occurred prior to diagnosis when cancer was progressing or already developed. Substantial weight loss prior to diagnosis could diminish weight loss thereafter, and along with the short-term follow-up, account for the lower than expected percentage of subjects that developed cachexia (i.e., 28% in the present study vs 50% anticipated from previous data provided elsewhere). Finally, this study consisted of a retrospective analysis of available data collected from standard of care procedures at a single institution. Future prospective research with larger sample sizes would benefit from extending beyond the analysis of routine, standard of care clinic data and to confirm that weight loss is caused by cachexia and not other factors, that the loss in body weight is related to the decline in muscularity and function, and to concomitantly identify other systemic inflammatory biomarkers that associate with cachexia, such as C-reactive protein, various interleukins or cytokines, angiotensin II, and neutrophil-derived proteases.

## Conclusion

In this investigation, we provide novel data demonstrating that an elevation in the NLR associates with greater weight loss and cachexia in subjects with advanced colon, lung, or prostate cancer. There was also a tendency for an elevation in the NLR to associate with lower serum 25(OH)D concentrations. Based on these findings, we conclude that an elevation in systemic inflammation associates with greater weight loss and cachexia, and potentially, lower serum 25(OH)D concentrations in patients with colon, lung, or prostate cancer. These findings could provide the rationale to identify and stratify cancer patients at risk for excessive weight loss and cachexia based on a readily-available and routine biomarker of systemic inflammation, the NLR, in future investigations.

## Supplementary information

Supplementary information
