# Supplementary material for: An elevated neutrophil-to-lymphocyte ratio associates with weight loss and cachexia in cancer
Source: Sci Rep. 2020 May 5;10:7535. doi: 10.1038/s41598-020-64282-z (PMC7200806; doi:10.1038/s41598-020-64282-z)
Supplement: Supplementary file 1 — Supplementary information [file 41598_2020_64282_MOESM1_ESM.docx]

**SUPPLEMENTAL FIGURE AND TABLE**

**Title:**

An elevated neutrophil-to-lymphocyte ratio associates with weight loss and cachexia in cancer

**Authors:**

Tyler Barker^1,2*^, Gail Fulde^1^, Bryce Moulton^1^, Lincoln D. Nadauld^1,3^, and Terence Rhodes^1^

**Affiliations:**

^1^ Precision Genomics, Intermountain Healthcare, St. George, UT 84790 USA

^2^ Department of Nutrition and Integrative Physiology, University of Utah, Salt Lake City, UT 84112 USA

^3^ School of Medicine, Stanford University, Stanford, CA USA 94305

***Corresponding Author:**

Tyler Barker, PhD

Intermountain Precision Genomics – Cancer Research Clinic

5121 S. Cottonwood Street, Building #2, Suite #610

Murray, UT 84107 USA

Phone: +1-801-507-3653

Fax: +1-801-507-3640

Email: [tyler.barker@imail.org](mailto:tyler.barker@imail.org)

**Supplemental Figure.** The NLR as a function of vitamin D status. The NLR was not significantly different between vitamin D status groups. Def, vitamin D deficient (n = 7); Insuff, vitamin D insufficient (n = 6); Suff, vitamin D sufficient (n = 37). Data presented as mean (SD).

| **Supplemental Table**. Serum 25(OH)D concentrations before and after disease diagnosis and treatment | | | | | |
| --- | --- | --- | --- | --- | --- |
|  | Disease Diagnosis | |  | Treatment | |
|  | Before (n = 13) | After (n = 37) |  | Before (n = 36) | After (n = 14) |
| Serum 25(OH)D, ng/mL | 34.0 (26.5) | 35.0 (17.5) |  | 35.0 (22.5) | 33.5 (17.0) |
| Data presented as median (interquartile range) | |  |  |  |  |
